# Daily Consumption of Unprocessed/Minimally Processed Foods and Its Relationship with Serum Phosphorus, CD3+, and CD45+ Cell Counts in People Living with HIV: A Cross-Sectional Descriptive Study

**DOI:** 10.3390/medsci14010141

**Published:** 2026-03-18

**Authors:** Kaila Souza Gomes Carvalho, David Michel de Oliveira, Mayara Bocchi, Fábio Morato de Oliveira, Eduardo Vignoto Fernandes

**Affiliations:** 1 Laboratory of Immunometabolism, Nutrition and Exercise (LABINE), Federal University of Jataí, Jataí 75801-615, Brazil; kaila.carvalho@discente.ufj.edu.br (K.S.G.C.); profdoliveira@ufj.edu.br (D.M.d.O.); mayara.fernandes@ufj.edu.br (M.B.); 2 Laboratory of Human and Medical Genetics, Federal University of Jataí, Jataí 75801-615, Brazil; fabiomorato@ufj.edu.br

**Keywords:** micronutrients, healthy eating, biomarkers, trace elements

## Abstract

Objectives: To investigate the potential associations between the daily consumption of unprocessed/minimally processed foods and serum phosphorus levels, CD3+, and CD45+ cell counts in clinically stable people living with HIV (PLHIV). Methods: This is a descriptive cross-sectional study. A total of 92 PLHIV of both sexes participated. Sociodemographic information, physical activity level, anthropometric and body composition data, dietary habits, and blood samples were collected. Results: The mean age of participants was 43.0 ± 12.0 years, with a body mass index of 26.5 ± 6.3 kg/m^2^. The majority were male (60.8%), single (64.1%), had low educational attainment (55.4%), were non-smokers (64.1%) and did not consume alcoholic beverages (51.1%), and were physically active (70.7%). A positive association was observed between the daily consumption of unprocessed/minimally processed foods and serum phosphorus levels (*p* = 0.01), as well as CD3+ (*p* = 0.04) and CD45+ (*p* = 0.04) cell counts. Furthermore, positive correlations were identified between this dietary pattern and serum phosphorus (*p* = 0.001; r = 0.33) and the percentages of CD3+ (*p* = 0.03; r = 0.21) and CD45+ (*p* = 0.03; r = 0.22). Conclusions: The present study suggests that habitual consumption of unprocessed/minimally processed foods is positively associated with serum phosphorus levels, CD3+, and CD45+ cell counts in PLHIV. While these associations do not imply causality or enhanced antiviral immunity, they highlight the potential role of diet quality in the metabolic and immunological maintenance of stable patients.

## 1. Introduction

Evidence from the literature indicates that the consumption of fresh or minimally processed foods has positive effects on health and quality of life [1]. The most well-known foods in these categories include vegetables, legumes, fruits, cereals, and meats. They are rich in nutrients and essential for maintaining the body’s basic functions and are also associated with a lower risk of mortality from non-communicable chronic diseases such as cancer, diabetes, hypertension, dyslipidemia, and cardiovascular diseases [2,3,4].

Dietary nutrients are classified into macronutrients and micronutrients. Macronutrients—carbohydrates, lipids, and proteins—are responsible for providing energy to sustain bodily functions. Micronutrients, which include vitamins and minerals, are essential substances for the body’s functioning, contributing alongside macronutrients to carbohydrate-to-energy conversion, oxygen transport, and DNA and protein synthesis [5].

Phosphorus, the focus of this study, is necessary for energy production in the form of high-energy phosphates (ATP) and functions in all cells of the body. Additionally, it plays a key role in maintaining the integrity of cell membranes, energy metabolism, and pH balance [6]. Given its significant functional importance, phosphorus must be consistently present in the diet. As foods such as meats, milk, grains, and seeds contain high concentrations of phosphorus (an average of 200 mg/100 g), replenishment is generally easy, thereby reducing the risk of deficiency [7].

The human immunodeficiency virus (HIV) causes chronic immune system activation and inflammation in infected individuals, leading to disease progression [8]. However, with proper use of antiretroviral therapy (ART), viral load decreases by inhibiting viral replication [9] and, consequently, the rate of HIV transmission is reduced [10]. Nonetheless, one side effect of ART is the onset of non-HIV-related comorbidities such as dyslipidemia, abdominal obesity, and cardiovascular disease, which may progress to metabolic syndrome [11]. These comorbidities compromise both the quality of life and longevity of people living with HIV (PLHIV) [12,13].

Thus, in addition to the immunometabolic alterations caused by infection and/or ART, PLHIV must constantly maintain an active immune system, which increases energy expenditure and raises phosphorus requirements [14,15,16,17]. Furthermore, PLHIV often have reduced digestive and absorptive capacity, leading to malnutrition and disease progression [18]. Studies have shown that PLHIV experience both quantitative and qualitative changes in gut microbiota, with such alterations causing tissue inflammation, mucosal damage, and immune activation [19,20,21,22], conditions that negatively impact nutrient absorption.

In addition to phosphorus, which contributes to sustaining immune system energy demands, attention should also be given to a membrane protein known as CD45+. This molecule is exclusively present in nucleated cells of the hematopoietic system [23] and plays an important role in combating infections, autoimmune diseases, and cancers [23,24,25]. CD45+ acts in CD4+ T cells by aiding the activation of lymphocyte-specific protein tyrosine kinase (Lck), which is responsible for phosphorylating the T cell antigen receptor complex (TCR) [26,27,28] and simultaneously regulates the termination of this same process [27]. In this way, CD45 can regulate T cell responses, either activating or inhibiting them as needed to detect and respond to antigens [27]. This molecule is directly affected by HIV, as PLHIV—particularly those not receiving ART—tend to have reduced levels of CD45+, a condition that directly impacts the patients’ immune protection [29].

Another regulatory protein is CD3+, which is part of the T lymphocyte receptor complex and is involved in signal transduction [30]. One study showed that CD3+ functions as an anti-HIV factor during the first month of infection, a period in which the molecule is found in large quantities. As the clinical condition progresses, its effectiveness decreases; however, CD8+ T cells with high CD3+ expression demonstrate superior viral control [31]. While CD4+ T-cell recovery and the CD4/CD8 ratio are the primary clinical gold standards for monitoring HIV progression, there is a growing interest in understanding how lifestyle factors, such as diet, relate to the broader immunological profile. In this study, CD3+ and CD45+ cell counts were selected as primary indicators to provide a comprehensive view of the pan-T cell population and total leukocyte homeostasis, respectively. CD3+ serves as a fundamental marker for the entire T-cell lineage, reflecting the overall adaptive immune reservoir, while CD45+ (the leukocyte common antigen) is essential for signal transduction and the regulation of immune cell activation. By focusing on these pan-markers, we aimed to explore whether dietary quality is associated with the maintenance of the systemic immune environment in PLHIV who have already achieved clinical stability under ART.

Overall, HIV progression can be influenced by nutritional imbalances involving micronutrients [32,33]. Therefore, in addition to ART, monitoring the nutritional status of PLHIV is crucial to preventing the infection from progressing to acquired immunodeficiency syndrome (AIDS) and increasing patient mortality risk [15,34,35,36,37]. Consequently, assessing and studying the diets of PLHIV is essential for providing more individualized patient care, ensuring that health and quality of life are preserved as effectively as possible [38].

Based on the nutritional benefits of a diet rich in whole foods, we hypothesized that a higher intake of unprocessed or minimally processed foods is positively associated with increased CD3+ and CD45+ cell counts and maintained serum phosphorus levels in PLHIV. Therefore, the aim of the present study was to investigate the potential associations between the daily consumption of unprocessed or minimally processed foods and serum phosphorus levels, CD3+, and CD45+ cell count levels in clinically stable PLHIV.

## 2. Materials and Methods

### 2.1. Study Design and Data Collection Site

This study employed a cross-sectional design with a quantitative approach and was prepared in accordance with the recommendations of the STROBE (Strengthening the Reporting of Observational Studies in Epidemiology) guidelines for observational studies [39]. Data collection was conducted at the Testing and Counseling Center—CTA and Specialized Care Service—SAE (CTA/SAE), located in Jataí, Goiás, Brazil.

### 2.2. Participant Recruitment and Sample Size Calculation

People living with HIV (PLHIV) were invited to participate in the study when they attended the CTA/SAE for routine laboratory tests. Participants were selected by convenience sampling, according to pre-established inclusion and exclusion criteria. Researchers approached PLHIV in the waiting room, and those expressing interest were invited to a private room for further explanation and to sign the informed consent form.

Sample size was calculated using the G*Power^®^ software, version 3.1.9.2 (Institute of Experimental Psychology, Düsseldorf, Germany), with type I and type II errors set at α = 0.05 and β = 0.05, respectively, to detect an effect size of 0.40 or greater. Based on this calculation, data collection from a minimum of 59 participants was required. In total, 92 PLHIV took part in the assessments, conducted between January and November 2024. The study was approved by the Research Ethics Committee (CEP) of the Federal University of Jataí (approval no.: 6.390.246; CAAE 71663823.2.0000.0187) on 5 October 2023. After CEP approval, the project was registered with the Brazilian Clinical Trials Registry (ReBEC) under number RBR-10zr5mjp (https://ensaiosclinicos.gov.br/rg/RBR-10zr5mjp), accessed on 22 May 2024.

### 2.3. Inclusion and Exclusion Criteria

Patients of both sexes diagnosed with HIV and receiving care at the CTA/SAE in Jataí for a minimum of six continuous months of treatment with the following therapeutic regimen were included in the study: dolutegravir 50 mg, tenofovir 300 mg, and lamivudine 300 mg. Exclusion criteria comprised PLHIV under the age of 18; women who were pregnant and/or lactating; and individuals who did not complete the data/sample collection phases or withdrew their consent during the study period.

### 2.4. Procedures and Instruments

Sociodemographic data (education level, marital status, age, sex, alcohol and tobacco use, and clinical information), physical activity level, dietary intake, blood samples, and anthropometric measurements were collected from PLHIV.

#### 2.4.1. Physical Activity Level (PAL)

PAL was assessed using the International Physical Activity Questionnaire—Short Form (IPAQ-SF). The IPAQ-SF classifies individuals as very active, active, insufficiently active, or sedentary [40]. For data presentation, participants were categorized as active (very active + active) or inactive (insufficiently active + sedentary), as proposed by [41].

#### 2.4.2. Dietary Intake Assessment

Dietary intake was evaluated using a questionnaire based on the classification of foods by degree and nature of processing, as defined in the Brazilian Dietary Guidelines [42]. Examples of foods were listed and categorized into: (i) unprocessed or minimally processed foods; (ii) oils, fats, salt, and sugar; (iii) processed foods; and (iv) ultra-processed foods. Participants reported the frequency of consumption, and only unprocessed or minimally processed foods were considered in this study.

To estimate the frequency of unprocessed or minimally processed food consumption, participants were asked about the intake of fresh or packaged, fractionated, chilled, or frozen vegetables, fruits, and tubers; rice; corn (grain or on the cob), wheat, and other cereal grains; beans, lentils, chickpeas, and other legumes; fresh or dried mushrooms; dried fruits; pasteurized fruit juices with no added sugar or other substances; nuts and oilseeds without added salt or sugar; manioc, corn, or wheat flours and pasta made from these flours and water; beef, pork, poultry, and fresh, chilled, or frozen fish; pasteurized, ultra-pasteurized (‘long-life’), or powdered milk; unsweetened yogurt; eggs; tea; coffee; and drinking water. Reported frequencies were converted into daily frequency equivalents as follows: every day = 1; 5–6 times/week = 0.79; 2–4 times/week = 0.43; once/week = 0.14; 1–3 times/month = 0.07; and never or rarely = 0 [43].

#### 2.4.3. Blood Collection and Hematological Analyses

Participants were instructed to fast for 8–12 h prior to blood collection. Peripheral blood was drawn from the cubital region (median, cephalic, or basilic veins) using a vacuum collection system. Samples were sent to the laboratory for processing and analysis, including complete blood count, biochemical profile, viral load, and counts of CD3+, CD4+, CD8+, and CD45+ cells.

The complete blood count was performed using the automated hematology analyzer Sysmex XN-350 (Sysmex, Kobe, Japan) with whole blood samples. The biochemical profile was analyzed using the automated Dimension^®^ EXL™ 200 analyzer (Siemens Healthcare Diagnostics Inc., Erlangen, Germany) with serum samples, applying the LOCI^®^ advanced chemiluminescence method. Tests included lipid profile, glucose, liver enzymes, creatinine, amylase, urea, sodium, potassium, phosphorus, and ionized calcium.

Plasma viral load was quantified using the cobas^®^ 5800 system (Roche Diagnostics, Indianapolis, IN, USA) with real-time PCR for HIV-1. Counts of CD3+, CD4+, CD8+, and CD45+ cells in whole blood were performed by flow cytometry using the BD FACSVia™ system with BD Multitest™ reagents.

#### 2.4.4. Anthropometric Assessment and Body Composition

Body mass was measured using a calibrated analog scale (Welmy^®^, Santa Bárbara d’Oeste, SP, Brazil) with 100 g precision. Height was measured by a stadiometer attached to the scale, with 0.5 cm precision. Body mass index (BMI) was calculated as weight (kg) divided by height squared (m^2^) [44]. Waist and hip circumferences were measured using a Sanny^®^ anthropometric tape, and the waist-to-hip ratio was calculated.

### 2.5. Statistical Analysis

Initially, data were tested for normality using the Kolmogorov–Smirnov test. Parametric data were expressed as mean ± standard deviation, and non-parametric data as median and interquartile range (25–75%). Categorical variables were expressed as absolute values and percentages. Fisher’s exact test was used for association analyses, with odds ratios (OR) and 95% confidence intervals (95% CI) reported when differences were observed. For the association analysis, the variables CD3+ and CD45+ cell counts were dichotomized into ‘high’ and ‘low’ categories using the median as the cutoff point. This approach was adopted due to the absence of established clinical consensus thresholds for these specific pan-markers within the context of immunologically stable PLHIV on long-term ART. The use of the median ensures a balanced distribution of the sample, providing sufficient statistical power to compare groups with higher versus lower cellular reservoirs within the distribution of this specific cohort. Spearman’s correlation test was applied to examine relationships between dietary intake and other study variables. Statistical analyses were conducted using GraphPad Prism version 9.5.1, with a significant threshold of *p* < 0.05.

## 3. Results

The anthropometric, dietary, clinical, and sociodemographic characteristics of the participants are summarized in Table 1. The sample consisted of adults with a mean age of 43.0 ± 12.0 years who, on average, presented excess body weight, with a mean BMI of 26.5 ± 6.3 kg/m^2^, and central adiposity evidenced by a waist circumference of 89.4 ± 15.4 cm and a waist-to-hip ratio of 0.92 ± 0.16. Participants reported consuming fresh or minimally processed food almost daily (0.96 ± 0.15). On average, individuals had been living with HIV for more than nine years (113.8 ± 72.1 months) and had been on antiretroviral therapy for approximately eight years (96.8 ± 61.5 months). Most individuals were male (60.8%), single (64.1%), and had low educational attainment (≤9 years of schooling: 55.4%). The majority did not smoke (64.1%), did not consume alcoholic beverages (51.1%), and were classified as physically active (70.7%).

Table 2 summarizes the median values and interquartile ranges of the hematological and biochemical parameters of the participants. Overall, individuals living with HIV demonstrated results within the established reference ranges for all indicators assessed. Of relevance, the viral load was undetectable in most participants, and median CD4 counts exceeded 500 cells/mm^3^—findings that reflect effective immune reconstitution and are indicative of a favorable clinical status in PLHIV.

**Figure 1:**
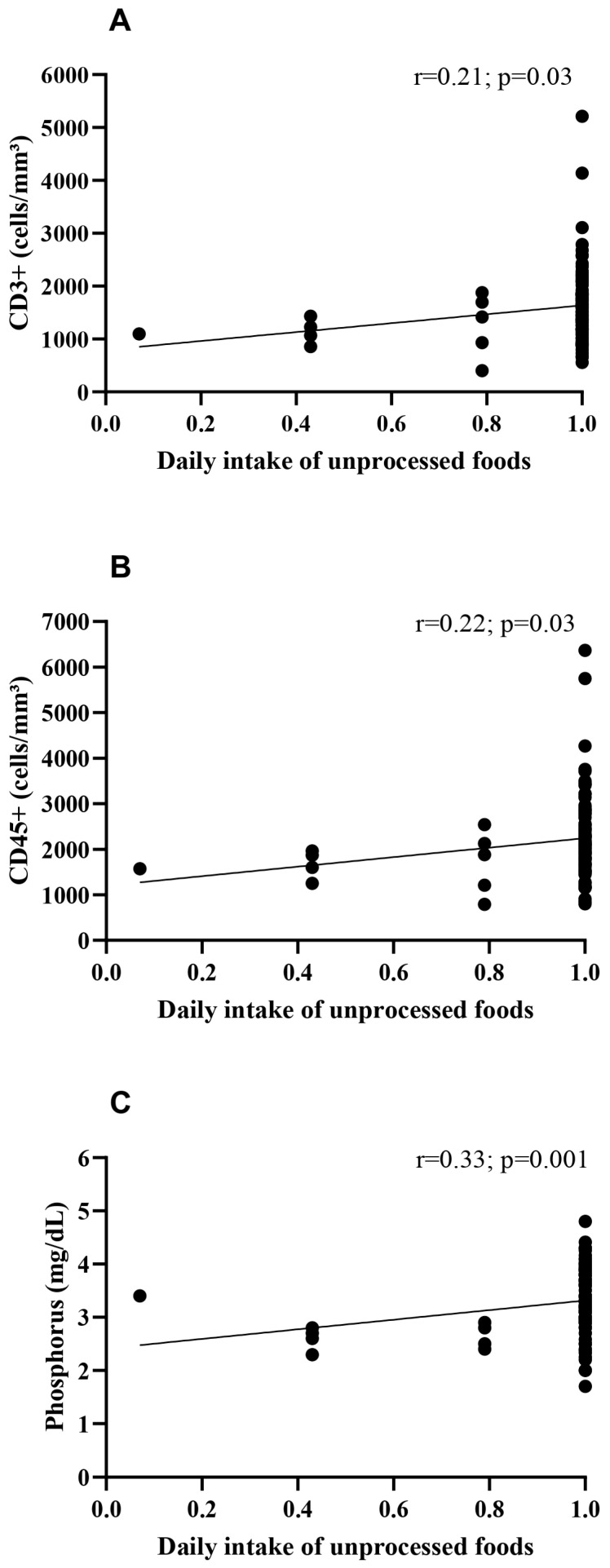
Correlation between the daily consumption of unprocessed or minimally processed foods and immunological and biochemical parameters in people living with HIV (PLHIV). (**A**) Positive correlation between daily consumption and CD3+ cell counts. (**B**) Positive correlation between daily consumption and CD45+ cell counts. (**C**) Positive correlation between daily consumption and serum phosphorus levels. These findings indicate that higher intake of fresh or minimally processed foods is associated with more favorable immunological and biochemical profiles in PLHIV.

Table 3 presents the associations between daily consumption of unprocessed or minimally processed foods and immune cells (CD4+, CD8+, CD3+, and CD45+) as well as micronutrients (sodium, potassium, phosphorus, and ionized calcium). A positive association was observed between daily consumption of unprocessed or minimally processed foods and CD3+ (*p* = 0.04), CD45+ (*p* = 0.04), and phosphorus (*p* = 0.01). No significant differences were found for the other variables analyzed (*p* > 0.05). Furthermore, when correlating the daily consumption of unprocessed or minimally processed foods with CD3+ (Figure 1A, *p* = 0.03; r = 0.21), CD45+ (Figure 1B, *p* = 0.03; r = 0.22), and phosphorus (Figure 1C, *p* = 0.001; r = 0.33), a positive correlation was also observed, indicating that people living with HIV (PLHIV) who consume higher amounts of natural foods tend to present higher levels of these cells and phosphorus. No correlations were observed for the remaining variables, such as CD4+, CD8+, and hematological parameters (*p* > 0.05).

## 4. Discussion

The present study aimed to investigate the potential associations between daily consumption of unprocessed or minimally processed foods serum phosphorus levels, CD3+, and CD45+ cell counts levels in clinically stable PLHIV. The findings indicate that the hematological, biochemical, and immunological parameters observed are within reference ranges, reflecting the clinical stability of this cohort, which is characterized by long-term adherence to ART and successful immune reconstitution. In this context, our study examines variations within a clinically stable population rather than disease progression or severe immune dysfunction [45]. The use of these medications, together with appropriate nutrition, plays a crucial role in maintaining patient health [46]. PLHIV experiencing malnutrition tends to progress to AIDS more rapidly due to compromised immunity. In contrast, well-nourished PLHIV are better equipped to combat disease [15,47]. This is attributable to the fact that certain micronutrients (minerals and vitamins) modulate the molecular responses of immune system cells during an infectious process, activating both T and B cells [48].

The findings of this study showed that PLHIV presented blood profiles—including viral load, complete blood count, biochemical profile, and CD3+, CD4+, CD8+, and CD45+ cell counts—within normal ranges. There is strong evidence that this stability may be attributed to continuous ART adherence for at least six months [49]. These patients also demonstrated attention to dietary habits, incorporating healthy foods into their daily diet—an essential factor in promoting health and preventing potential HIV-related complications [50,51,52]. Moreover, most participants were physically active, which is another factor that contributes to overall health [53]. Smoking was infrequently reported, and less than half consumed alcoholic beverages. Such data indicate positive health prospects for PLHIV, as smoking can reduce life expectancy, preventing patients from achieving the same longevity as HIV-negative individuals [54]. This also applies to alcohol consumption, since the literature establishes that excessive intake contributes to disease progression [55].

The results of the present study demonstrate that daily consumption of unprocessed or minimally processed foods is positively associated with the overall health of PLHIV. These findings are relevant, as healthy eating supports the prevention and management of diseases and promotes overall health maintenance [56]. This association is biologically plausible, given that unprocessed foods contain nutrients and bioactive compounds responsible for sustaining an efficient immune response [57]. This is particularly important for PLHIV, as ART can lead to metabolic imbalances, such as lactic acidosis, bone demineralization, and lipodystrophy, which may impair immune response effectiveness [58,59]. The literature indicates that a balanced diet can help mitigate adverse effects associated with long-term ART, such as dyslipidemia [60]. While our data do not show a direct impact of diet on ART effectiveness itself, they support the role of nutrition as a complementary factor in the metabolic management of PLHIV.

Both the association and correlation between daily consumption of unprocessed or minimally processed foods and serum levels of phosphorus, CD3+, and CD45+ underscore the importance of a healthy diet, even for PLHIV. The significance of phosphorus lies in its role in ATP production [7,61,62] and in maintaining homeostasis [7,61]—conditions essential for sustaining an active immune system. Conversely, low levels of this element may lead to reduced intestinal absorption, intracellular shifts of phosphorus from the blood, and depletion of intracellular phosphate [63]. While not directly measured in this study, the observed association between serum phosphorus and dietary patterns leads us to hypothesize a potential link involving cellular energy metabolism. However, further research involving direct measurements of cellular metabolism and cytokines is required to confirm if these serum variations translate into functional immune differences in PLHIV.

Consumption of natural foods also exerts a substantial influence on CD3+—which is involved in T cell activation signaling and antigen recognition—and on CD45+, which is associated with the proper functioning of T and B lymphocytes [23,64,65]. Nutritional imbalances affecting the function and quantity of these molecules not only impair the body’s defense against the virus but also impact the overall health status of PLHIV, as a suppressed immune system cannot effectively control viral replication rates [66,67]. Thus, it becomes evident that PLHIV, in addition to ART, should pay close attention to dietary habits to avoid nutritional deficiencies, particularly phosphorus deficiency, given its essential role in energy production and in the synthesis of molecules such as CD3+ and CD45+, which are fundamental for immune system function. The positive association between unprocessed food intake and CD3+ and CD45+ cell counts suggests a relationship between diet quality and these pan-leukocyte markers. It is important to note, however, that since CD3+ and CD45+ are broad markers, these variations in clinically stable patients may not necessarily indicate enhanced antiviral immunity or superior viral control. Instead, they may reflect an overall well-maintained physiological state associated with better nutritional habits. It is essential to note that our cohort is composed of individuals who have achieved immune reconstitution and long-term viral suppression through ART. Therefore, our results regarding the relationship between diet and immune markers represent variations within a clinically stable population, and these findings should not be interpreted as evidence for the treatment of severe immune dysfunction or HIV-related disease progression.

This study has some limitations. First, its cross-sectional design precludes longitudinal monitoring of dietary habits and limits causal inferences. Second, we did not account for potential confounding variables—such as socioeconomic status, alcohol consumption, and physical activity intensity—which may independently influence both nutritional choices and biomarkers. Additionally, the exposure variable (daily consumption score) showed limited variability among participants, which may have attenuated the strength of the observed correlations and should be considered when interpreting the results. However, to mitigate potential biases and enhance statistical reliability, the study included a sample size nearly double the initial requirement, providing greater robustness to the identified associations within this clinically stable cohort. Furthermore, the novelty of this study lies in its integrated approach to the nutritional–immunological axis in PLHIV. Unlike most research that focuses on the deleterious effects of ultra-processed food consumption, this study is pioneering in demonstrating how a dietary pattern centered on unprocessed/minimally processed foods is positively associated with serum phosphorus—a key marker of cellular energy metabolism—and the maintenance of CD3+ and CD45+ T-cell counts. By bridging the gap between a specific food processing classification and objective biomarkers of immune recovery, our findings emphasize a more positive and practical nutritional perspective for the clinical management of chronic inflammation in this population.

The clinical applicability of these findings lies in the establishment of a clear, evidence-based framework for nutritional counseling in HIV care. By demonstrating a positive correlation between unprocessed food patterns and markers such as serum phosphorus and T cell counts, this study suggests that nutritional interventions should transition from generic caloric management to a focus on food quality and processing levels. In practice, this allows healthcare providers to implement a ‘food-first’ approach that is both cost-effective and sustainable, directly supporting the metabolic and immunological demands of patients undergoing long-term ART. Such an approach serves as a vital non-pharmacological adjunct to mitigate chronic inflammation and improve cellular energy status, offering a feasible strategy to enhance the long-term clinical prognosis and quality of life for PLHIV across diverse socioeconomic settings.

## 5. Conclusions

The present study examined the relationship between the consumption of unprocessed or minimally processed foods and their effects on blood micronutrient levels and body composition in PLHIV. The findings indicate that more frequent intake of unprocessed or minimally processed foods is positively associated with increased phosphorus levels—essential for energy metabolism—as well as the immunological markers CD3+ and CD45+, both of which are crucial for immune function.

In this study of immunologically reconstituted individuals, nutritional monitoring of PLHIV is essential for maintaining an adequate health profile. Although the observed correlations are modest and do not demonstrate a direct impact on disease progression or ART effectiveness, they underscore the importance of dietary quality as a complementary factor in the long-term care of PLHIV. Future longitudinal studies are needed to further clarify the nature of these associations.

## Figures and Tables

**Table 1:** Anthropometric, dietary, clinical, and sociodemographic profile of PLHIV.

**Continuous Variables**	**Mean ± SD**
Age (years)	43.0 ± 12.0
Height (m)	1.66 ± 0.08
Body mass (kg)	73.8 ± 18.5
BMI (kg/m^2^)	26.5 ± 6.3
Waist circumference (cm)	89.4 ± 15.4
Hip circumference (cm)	98.9 ± 18.0
WHR	0.92 ± 0,16
Daily consumption of fresh or minimally processed foods	0.96 ± 0.15
Time since HIV diagnosis (months)	113.8 ± 72.1
Duration of antiretroviral therapy (months)	96.8 ± 61.5
**Categorical Variables**	***n*** **(%)**
Sex	
Female	36 (39.2)
Male	56 (60.8)
Marital status	
With partner	33 (35.9)
Without partner	59 (64.1)
Education level (years of schooling)	
≤9 years	51 (55.4)
>9 years	41 (44.6)
Smoker	
Yes	33 (35.9)
No	59 (64.1)
Alcohol consumption	
Yes	45 (48.9)
No	47 (51.1)
Physical activity level (PAL)	
Active	65 (70.7)
Inactive	27 (29.3)

Legend: PLHIV, people living with HIV; SD, standard deviation; BMI, body mass index; WHR, waist-to-hip ratio; PAL, physical activity level.

**Table 2:** Profile of blood parameters in PLHIV.

Variables	Median (Quartile: 25–75%)	Reference Values
Viral load (copies/mL)	0 (0–53)	0 or undetectable
CD3+ (mm^3^)	1508 (1138–1853)	1025–2904
CD4+ (mm^3^)	553 (330–800)	540–1731
CD8+ (mm^3^)	807 (548–1169)	263–1189
CD45+ (mm^3^)	2058 (1617–2543)	1257–4104
Red blood cells (10^6^/mm^3^)	4.74 (4.43–5.05)	4.50–5.90
Hemoglobin (g/dL)	14.4 (13.3–15.1)	12.0–16.0
Hematocrit (%)	40.9 (39.1–43.8)	41.0–53.0
Leukocytes (mm^3^)	5680 (4555–6975)	3500–10,000
Neutrophils (%)	61 (53–67)	36–66
Lymphocytes (%)	30 (23–37)	25–45
Monocytes (%)	7 (5–8)	2–10
Platelets (10^6^/mm^3^)	220 (181–266)	130–500
Glucose (mg/dL)	86 (78–101)	60–99
Triglycerides (mg/dL)	115 (75–153)	<150
Total cholesterol (mg/dL)	176 (146–203)	<130
LDL (mg/dL)	100 (76–124)	<100
HDL (mg/dL)	44.5 (37.5–58.0)	>40
VLDL (mg/dL)	23 (16–31)	<30
AST/TGO (U/L)	24 (21–33)	10–40
ALT/TGP (U/L)	32 (19–45)	10–45
Urea (mg/dL)	31 (25–36)	18–50
Amylase (U/L)	69 (53–89)	25–125
Creatinine (mg/dL)	1.0 (0.84–1.16)	0.6–1.25
Sodium (mmol/L)	138 (137–140)	136–145
Potassium (mmol/L)	4.4 (4.2–4.7)	3.5–5.1
Phosphorus (mg/dL)	3.2 (2.9–3.7)	2.3–4.7
Ionized calcium (mg/dL)	1.26 (1.20–1.32)	1.12–1.32

Legend: CD, cluster of differentiation; LDL, low density lipoprotein; HDL, high-density lipoprotein; VLDL, very-low-density lipoprotein; AST, aspartate transaminase; ALT, alanine aminotransferase. The values are presented as median and interquartile range (25–75%).

**Table 3:** Association between daily consumption of unprocessed or minimally processed foods and immune system cells and micronutrients in PLHIV.

Variables	No	Yes	OR (95% CI)	*p*
	*n* (%)	*n* (%)		
CD4+ (mm^3^)				
≤550	05 (50.0)	41 (50.0)		0.99
>550	05 (50.0)	41 (50.0)	-	
CD8+ (mm^3^)				
≤800	06 (60.0)	39 (47.6)		0.51
>800	04 (40.0)	43 (52.4)	-	
CD3+ (mm^3^)				
≤1500	08 (80.0)	37 (45.1)	4.8 (1.0–23.5)	0.04
>1500	02 (20.0)	45 (54.9)		
CD45+ (mm^3^)				
≤2000	08 (80.0)	35 (42.7)	5.3 (1.1–25.9)	0.04
>2000	02 (20.0)	47 (57.3)		
Sodium (mmol/L)				
≤138	05 (50.0)	32 (40.0)		0.73
>138	05 (50.0)	48 (60.0)	-	
Potassium (mmol/L)				
≤4.4	06 (60.0)	39 (48.8)		0.73
>4.4	04 (40.0)	41 (51.2)	-	
Phosphorus (mg/dL)				
≤3.2	08 (88.9)	35 (43.8)	10.2 (1.3–116.3)	0.01
>3.2	01 (11.1)	45 (56.2)		
Ionized calcium (mg/dL)				
≤1.2	04 (44.4)	15 (23.4)	-	0.22
>1.2	05 (55.6)	49 (76.6)		

Legend: CD, cluster of differentiation; OR, Odds Ratio; IC95%, confidence interval.

## Data Availability

The original contributions presented in this study are included in the article. Further inquiries can be directed to the corresponding author.

## Abbreviations

ATP	Adenosine Triphosphate
CD3	Cluster of differentiation 3
CD4	Cluster of differentiation 4
CD8	Cluster of differentiation 8
CD45	Cluster of differentiation 45
ART	Antiretroviral therapy
DNA	Deoxyribonucleic Acid
AIDS	Acquired immunodeficiency syndrome
HIV	Human immunodeficiency virus
PLHIV	People living with human immunodeficiency virus
STROBE	Strengthening the Reporting of Observational Studies in Epidemiology
CTA/SAE	Testing and Counseling Center and Specialized Care Service
CEP	Research Ethics Committee
ReBEC	Brazilian Clinical Trials Registry
PAL	Physical activity level
IPAQ-SF	International Physical Activity Questionnaire—Short Form
95% CI	95% confidence intervals
OR	Odds ratios
WHR	Waist-to-hip ratio
SD	Standard deviation
BMI	Body mass index
LDL	Low density lipoprotein
HDL	High-density lipoprotein
VLDL	Very-low-density lipoprotein
AST	Aspartate transaminase
ALT	Alanine aminotransferase
